# Anti-proliferative and cytotoxic activities of *Allium autumnale* P. H. Davis (Amaryllidaceae) on human breast cancer cell lines MCF-7 and MDA-MB-231

**DOI:** 10.1186/s12906-018-2105-0

**Published:** 2018-01-25

**Authors:** Ovgu Isbilen, Nahit Rizaner, Ender Volkan

**Affiliations:** 1 Biotechnology Research Center, Cyprus International University, Nicosia, 99258 Northern Cyprus via Mersin 10 Turkey; 2 Bioengineering Department, Faculty of Engineering, Cyprus International University, Nicosia, 99258 Northern Cyprus via Mersin 10 Turkey; 3 Faculty of Pharmacy, Cyprus International University, Nicosia, 99258 Northern Cyprus via Mersin 10 Turkey

**Keywords:** *Allium Autumnale*, MCF-7, MDA-Mb-231, Anti-cancer, Proliferation, Cytotoxicity

## Abstract

**Background:**

Natural products obtained from plants can be potent sources for developing a variety of pharmaceutical products. *Allium* species have been widely studied for their anti-cancer effects and presented promising results as potential anti-cancer agents. Breast cancer (BCa) is one of the most commonly diagnosed types of cancer in women. In this study, we aimed to investigate the anti-proliferative, cytotoxic and anti-metastatic effects of bulb and stem extracts from *Allium autumnale* P. H. Davis (Amaryllidaceae), an endemic *Allium* species to the island of Cyprus, in a comparative approach to weakly metastatic MCF-7 and strongly metastatic MDA-MB-231 breast cancer (BCa) cell lines.

**Methods:**

Possible cytotoxic, anti-proliferative and anti-metastatic effects of the *Allium* extracts on MCF-7 and MDA-MB-231 cells were tested using trypan blue exclusion, MTT and wound heal assays, respectively. Gas Chromatography Mass Spectroscopy (GC-MS) analysis was performed to determine the prominent medically important compounds in *Allium autumnale* bulb (AAB) and *Allium autumnale* stem (AAS) extracts. Student unpaired *t*-test or ANOVA followed by Newman-Keuls post hoc analysis (INSTAT Software) was used where appropriate.

**Results:**

Our results demonstrate that AAB extract (24, 48 and 72 h) exerts significant anti-proliferative effect on both MCF-7 and MDA-MB-231 cells where this effect for AAS extract was observed only at high (5000 and 10,000 μg/mL) concentrations. Cell viability experiments revealed that AAB extract incubations caused more cytotoxicity on both BCa cell lines compared to the AAS. In contrast, there was no effect on lateral motilities of either cell line.

**Conclusion:**

Overall, our studies demonstrated the anti-cancer activities associated with *Allium autumnale,* revealing it’s cytotoxic and anti-proliferative potential to be further utilized in in vivo studies.

## Background

Plants have historically been used for their health benefits and for their medicinal properties to treat diseases. World Health Organization (WHO) reports emphasize that most of the world’s population depend on traditional medicines for treatment purposes and many therapeutics owe their origin to medicinal plants [1]. *Allium* species have been widely used as therapeutic agents for centuries [2]. The most popular members of this family are the *Allium sativum* (garlic) and *Allium cepa* (onion) [3]. *Allium sativum* and *Allium cepa* are widely studied for their chemical composition and it was shown that they reserve various sulfur containing compounds, amino acids, vitamins, selenium and many antioxidants [3] and have various medically important properties including anti-microbial, anti-viral, anti-cancer, anti-oxidant, anti-hypertensive functions [4]. Experimental results pointing out the medical significance of these *Allium* species, catalyzed research on various other *Allium* species, making them attractive targets for research catapulting several therapeutic advances [3, 4].

Cancer is one of the leading causes of death especially in developed countries [5]. Breast cancer (BCa) is the leading cause of mortality among female cancer patients [6]. While it can often be treated with chemotherapy and surgical applications, the disease can easily relapse. Conventional treatment procedures and their side effects can be devastating for cancer patients and drastically reduce their quality of life which increase the demand for the development of novel approaches and complementary therapies to cancer treatment [7, 8].

The flora of the island of Cyprus reserves many understudied endemic plant species with potential therapeutic values. Our team have previously carried out anti-cancer studies on *Allium willeanum,* an endemic species belonging to the *Allium* family (O. Isbilen et al., unpublished data). It was shown that *Allium willeanum* has anti-proliferative, cytotoxic and anti-metastatic activity on MCF-7 and MDA-MB-231 BCa cells. The aim of this study is to investigate the anti-proliferative, cytotoxic and anti-metastatic effects of a related species, *Allium autumnale* P.H. Davis (Amaryllidaceae)*,* also endemic to the island of Cyprus [9]. Our results presented here demonstrate the anti-proliferative and cytotoxic anti-cancer activities associated with the never before studied species *Allium autumnale,* exerted on both strongly and weakly metastatic breast cancer cell lines. Overall, our studies point out that understudied *Allium* species endemic to Cyprus can be used for development of complementary and alternative drugs to maintain or improve diseases including cancer.

## Methods

### Plant sample collection

Fresh *Allium autumnale* plants were collected from North Cyprus Beşparmak (Pentadaktylos) Mountain range on September 2016 and were identified by Prof. Dr. Mehmet Koyuncu, Cyprus International University, Faculty of Pharmacy, Chair of Pharmaceutical Botany Department. A specimen of the collected *Allium autumnale* was deposited in the Cyprus International University Public Herbarium.

### Extraction of plant material

AAB and AAS samples were separated, chopped and air dried at room temperature. Mixer grinder was used to powder the dried plant samples. The extraction was performed by mixing powdered plant samples with 95% ethanol as previously carried out [10, 11]. Three back to back macerations of mixture were performed at the room temperature for 8 h and ethanolic extracts were filtered through Whatman No^o^1 filter paper. Filtrates were concentrated using Rotary-evaporator (Heidolph, Germany) at 40 °C. AAB and AAS extracts were labelled and stored at 4 °C for further analysis.

### Cell line and cell culture

Strongly metastatic BCa cell line MDA-MB-231 and weakly metastatic BC cell line MCF-7 were obtained from Imperial College London, UK (courtesy of Prof. Dr. Mustafa Djamgoz). Both cell lines were grown in Dulbecco’s Modified Eagle Medium (DMEM) (Gibco by Life Technologies™, USA) supplemented with 4 mmol/L L-glutamine and 10% fetal bovine serum (FBS) and incubated at 37 °C, 5% CO_2_ and 100% relative humidity. MCF-7 and MDA-MB-231 BCa cells were used for the experiments when they reached 80–100% confluence [12].

### Methyl-thiazolyl tertrazolium (MTT) assay

Proliferation of MDA-MB-231 and MCF-7 cells was studied by colorimetric 3-(4,5-dimethylthiazol-2-yl)-2,5-diphenyltetrazolium bromide (MTT) assay previously described by Fraser, et al. [12]. Both MCF-7 and MDA-MB-231 cells were plated with 3 × 10^4^/mL density and allowed to settle overnight before the AAB and AAS extract treatments were applied. Cells were incubated for 24, 48 and 72 h with the AAB and AAS treatment. Control group received 1 mL DMEM only. Multi-well plate reader (ELx800, Biotek Instruments) was used for performing the measurements at 490 nm. All experiments were performed at least in triplicates [12].

### Trypan blue exclusion assay

Trypan blue exclusion assay was used to determine the cytotoxicity of AAB and AAS extracts on MDA-MB-231 and MCF-7 BCa cells. Cells were plated with 3 × 10^4^/mL density and allowed to incubate overnight to settle before application of extract treatment. Cells were incubated for 24, 48 and 72 h with the AAB and AAS treatment. Control group received 1 mL DMEM only. Cells were viewed under inverted microscope (Leica, Germany) and % viability of cells was calculated from 30 randomly selected areas. All experiments were performed at least in triplicates [12].

### Lateral motility (wound heal) assay

The effect of AAB and AAS extracts on cell motility was demonstrated by using ‘wound heal’ assays. Cells were plated with 4 × 10^5^/mL density per 35 mm diameter cell culture dish and incubated for 24 h a 37^o^ C 5% CO_2_. Three wounds were created by using 200 μL Gilson pipette tips. The media was replaced with fresh DMEM containing AAB and AAS treatment with increasing concentrations, wounds were photographed using a digital microscope camera (Leica, Germany). After 24 h of incubation with AAB and AAS extracts, wounds were re-measured by using ImageJ software. Each lateral motility experiment was carried out in triplicates. Motility index (MoI) was calculated using formula: MoI = 1-(W_t_-W_0_) [13].

### Gas chromatography mass spectroscopy (GC-MS) analysis

GC-MS analysis was performed to investigate the molecular composition of AAB and AAS extracts according to the method as previously described by Lekshmi et al. [14]. Filtered ethanolic extracts of AAB and AAS extracts were analyzed using a Shimadzu, GCMS*-*QP2010 Plus system. Helium was used as carrier gas in the constant flow mode at 1 mL/min. The initial oven temperature was 50 °C and was maintained at this temperature for 2 min and then gradually increased up to 280 °C at the rate of 5 °C/min and maintained for 9 min. Injection port temperature was 250 °C and helium flow rate as 1 mL/min. Separation was achieved by TRB-5MS column about 30 m long 0.25 μm thickness and 0.25 μm diameter. Quadrupole Mass Detector was employed to detect compounds when they were vented from the column. Temperature of the detector was 250 °C. WILEY7.LIB MS data library was used to analyze the spectrum and identify the compounds detected [14].

### Statistical analysis

All experiments were carried out at least in triplicates and data obtained presented as means ± SEM. Where appropriate, Student unpaired *t*-test or one-way ANOVA (INSTAT Software) followed by Newman-Keuls post hoc analysis was performed. Results were considered significant at *p* < 0.05 (*) or *p* < 0.01 (**).

## Results

### Anti-proliferative effects of AAB and AAS extracts on MDA-MB-231 and MCF-7 cell lines

MTT assay was performed to determine the anti-proliferative effects of AAB and AAS on strongly and weakly/non-metastatic BCa cells, MDA-MB-231 and MCF-7, respectively.

**Fig. 1:**
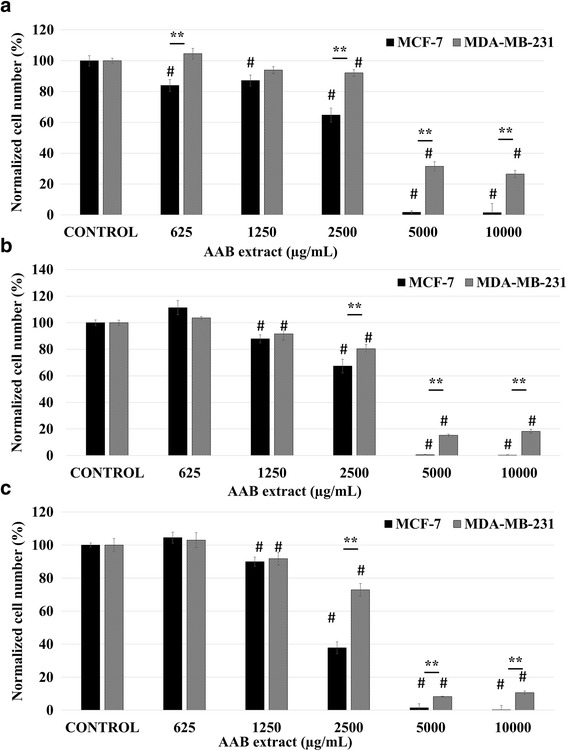
AAB extract causes significant anti-proliferative effect on MCF-7 and MDA-MB-231 cells. Concentration-effect curves of AAB extract treatment 625–10,000 μg/mL for 24 h (**a**), 48 h (**b**), and 72 h (**c**) in MCF-7 and MDA-MB-231 cells. Normalized cell number was calculated by using measurements from six independent MTT assays. Data is represented as mean ± S.E.M. Statistical significance: #, *p* < 0.05 vs control according to ANOVA followed by Newman-Keuls post hoc analysis. *, *p* < 0.05 and **, *p* < 0.01 MCF-7 vs MDA-MB-231 according to Student’s *t-*test

Incubation of AAB extract with MCF-7 (24 h:98.5%; 48 h:99.68%; 72 h:99.77% decrease in 10,000 μg/mL vs control; *p* < 0.01; *n* = 6) and MDA-MB-231 (24 h:73.5%; 48 h:81.77%; 72 h: 89.39% decrease in 10,000 μg/mL vs control; *p* < 0.01; *n* = 6) cells reduced proliferation in both BCa cell lines. The reduction in proliferation was observed in a concentration and time dependent manner (Fig. 1) which became more obvious especially at higher (5000 and 10,000 μg/mL) concentrations. An initial proliferation decline was observed on MCF-7 cells upon addition of 625 μg/mL AAB extract at 24 h, however a recovery was observed at 48-72 h time points which was followed by a consistent but slow decline at 1250–2500 μg/mL and a strong decline at 5000 μg/mL onwards at all time points. This data suggests that at low concentrations, despite initially responding, the MCF-7 cells can recover from the anti-proliferative effects of the extract where a robust reduction is observed at higher concentrations. This is consistent with the cancer cells’ ability to compensate for/adapt to the prolonged applications of chemical interventions and drugs [15].

**Fig. 2:**
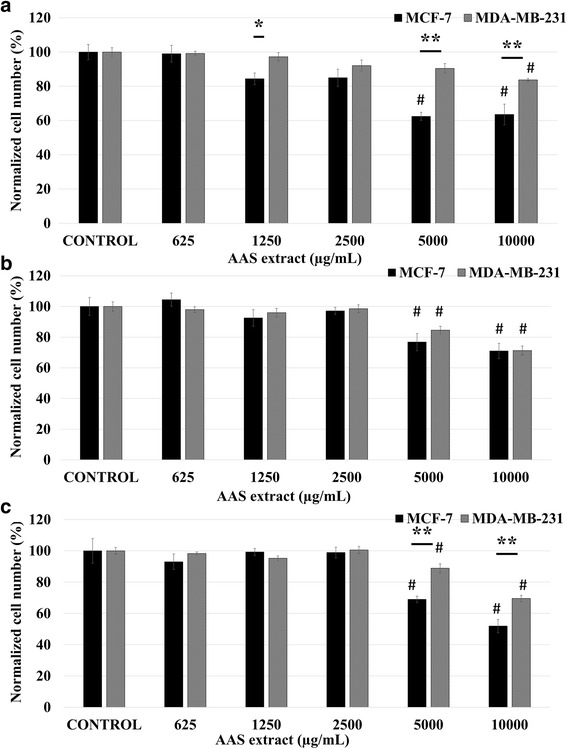
AAS extract causes significant anti-proliferative effect on MCF-7 and MDA-MB-231 cells. Concentration-effect curves of AAS extract treatment 625–10,000 μg/mL for 24 h (**a**), 48 h (**b**), and 72 h (**c**) in MCF-7 and MDA-MB-231 cells. Normalized cell number was calculated by using measurements from six independent MTT assays. Data is represented as mean ± S.E.M. Statistical significance: #, *p* < 0.05 vs control according to ANOVA followed by Newman-Keuls post hoc analysis. *, *p* < 0.05 and **, *p* < 0.01 MCF-7*vs* MDA-MB-231 according to Student’s *t-*test

AAS treatment also reduced cell proliferation in both MCF-7 (24 h: 36.49%; 48 h: 29.1%; 72 h: 48.1% decrease in 10,000 μg/mL vs control; *p* < 0.01; *n* = 6) and MDA-MB-231 cells (24 h: 16.27%; 28.74%; 72 h: 30.49% decrease in 10,000 μg/mL vs control; *p* < 0.01; *n* = 6). The observed reduction in proliferation rates of both cell lines is in a concentration and time dependent manner however the reduction in proliferation was less robust compared to the AAB treatment as the most significant results were observed at 5000 μg/mL onwards (Fig. 2).

MTT assay data have revealed that both AAB and AAS extracts are effective antiproliferative agents on MCF-7 and MDA-MB-231 BCa cells. The anti-proliferative effect of AAB extract treatment for all time periods was more potent on MCF-7 than MDA-MB-231 cells (Fig. 1). Similar pattern of anti-proliferative effect was also observed in both BCa cell lines treated with AAS extract albeit with reduced potency (Fig. 2).

### In vitro cytotoxic effects of AAB and AAS extracts on MDA-MB-231 and MCF-7 breast cancer cell lines

Trypan blue exclusion assay was performed to determine the impact of AAB and AAS treatment on the viability of MCF-7 and MDA-MB-231 BCa cells at for 24, 48 and 72 h time points. Increasing concentrations of AAB and AAS extract was applied for designated time periods (24, 48 and 72 h) on both cell lines. Results were comparatively analyzed for both MCF-7 and MDA-MB-231 BCa cells.

**Fig. 3:**
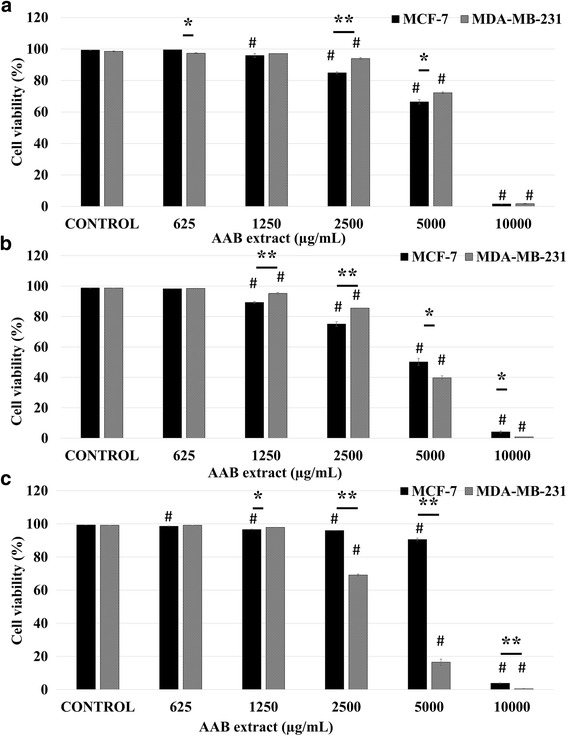
AAB extract significantly reduces viability of MCF-7 and MDA-MB-231cells. The % viability of breast cancer cells was determined by viability assay for 24 h (**a**), 48 h (**b**) and 72 h (**c**). Data represents mean ± S.E.M. of six independent experiments. Statistical significance: #, *p* < 0.05 vs control according to ANOVA followed by Newman-Keuls post hoc analysis. *p* < 0.05 and **, *p* < 0.01 MCF-7 vs MDA-MB-231 according to Student’s *t-*test

AAB extract treatment on both MCF-7 (24 h:97.72%; 48 h:94.58%; 72 h:95.5% decrease in viability; 10,000 μg/mL vs control; *p* < 0.01; *n* = 6) and MDA-MB-231 cells (24 h: 96.77%; 48 h: 97.89%; 72 h: 95.52%; decrease in viability; 10,000 μg/mL vs control; *p* < 0.01; n = 6) caused a significant reduction in cell numbers revealing the AAB extract’s cytotoxic effects on selected cancer cell lines in a concentration and time dependent manner. The most significant reduction was observed at the 48th hour time point on both cell lines, with a transient MCF-7 recovery at 72 h followed by a drastic decrease at 10000 μg/mL. Overall, both cell lines demonstrated significant reduction in viability, with lower viability values observed in MDA-MB-231 over time (Fig. 3).

**Fig. 4:**
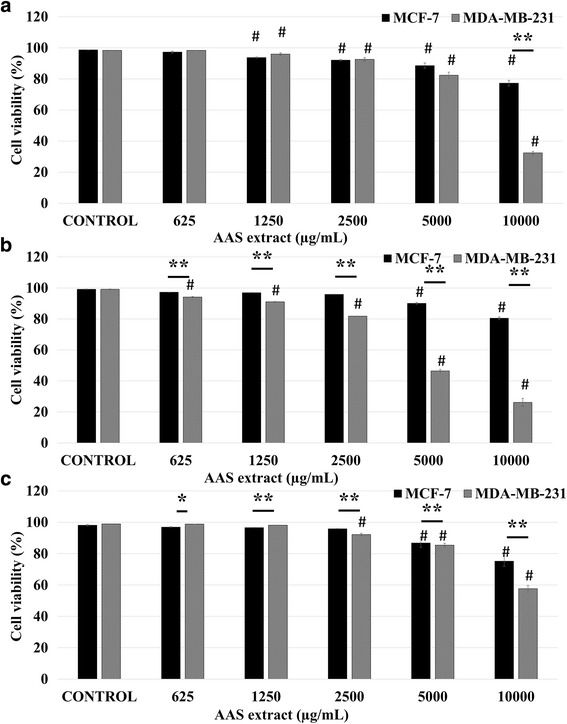
AAS extract significantly reduces viability of MCF-7 and MDA-MB-231cells. The % viability of breast cancer cells was determined by viability assay for 24 h (**a**), 48 h (**b**) and 72 h (**c**). Data represents mean ± S.E.M. of six independent experiments. Statistical significance: #, *p* < 0.05 vs control according to ANOVA followed by Newman-Keuls post hoc analysis.*, *p* < 0.05 and **, *p* < 0.01 MCF-7 vs MDA-MB-231 according to Student’s *t-*test

AAS extract treatment similarly reduced the cell numbers in a concentration and time dependent manner on MCF-7 (24 h: 21.4%; 48 h:18.65%; 72 h: 22.98% decrease in viability; 10,000 μg/mL vs control; *p* < 0.01; *n* = 6) and MDA-MB-231 cells (24 h: 65.36%; 48 h: 73%; 72 h: 41.35%; decrease in viability; 10,000 μg/mL vs control; *p* < 0.01; *n* = 6). A significant decrease in viability was observed at 2500 μg/mL on both cell lines which was followed by recovery of MCF-7 cells at 48 and 72 h whereupon, reduction in viability was observed at higher concentrations. However, MDA-MB-231 cells consistently continued to display lower viability at this concentration on, at all time points, revealing a more consistent and significant impact of AAS on this strongly metastatic cell line (Fig. 4).

Data obtained from viability assays revealed that AAB extract treatment exhibited significant cytotoxic effects on MCF-7 cells at 24 h and 48 h incubation time points, particularly at 2500, 5000 and 10,000 μg/mL concentrations (*p* < 0.01, *n* = 6), where significant cytotoxicity was observed on the highly metastatic MDA-MB-231 cells at 72 h incubation time points, specifically at 2500, 5000 and 10,000 μg/mL concentration (*p* < 0.01, *n* = 6) (Fig. 3). On the other hand AAS extract exhibited significant cytotoxicity especially on MDA-MB-231 cells compared to the MCF-7 cells at 5000 and 10,000 μg/mL concentration (*p* < 0.01, *n* = 6) (Fig. 4). When both extracts were compared, AAB extract exhibited comparatively more potent cytotoxicity on both MCF-7 and MDA-MB-231 cell lines for all time periods.

#### Blebbing

**Fig. 5:**
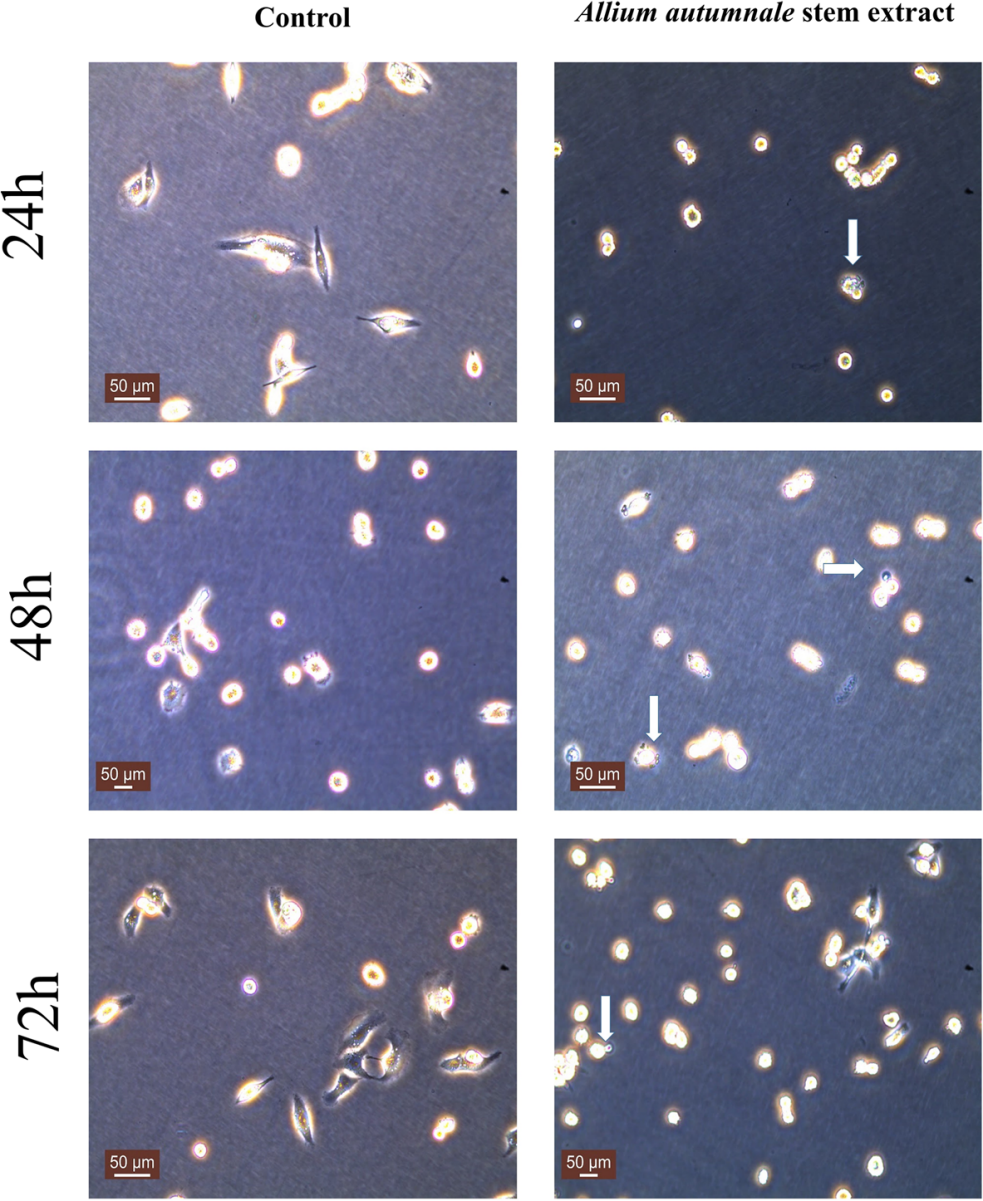
AAS extract induces membrane blebbing on MDA-MB-231 cells. Light microscope images (20×) show non treated (left panel) and 2500 μg/mL AAS extract treated (right panel). Arrows indicated plasma membrane blebbing after incubations with *Allium autumnale* stem extract. Scale Bars = 50 μm

Morphological investigation of BCa cells under inverted microscope (20× magnification) revealed formation of membrane blebs indicative of apoptotic mechanisms, on MDA-MB-231 cells treated with AAS extract (Fig. 5). No visible blebs were observed on the same cell line treated with the AAB extract. Interestingly, MCF-7 cells treated with the AAS extract did not show presence of any plasma membrane blebbing indicating the blebbing phenomenon to be specific to the strongly metastatic breast cancer cell line MDA-MB-231.

### Lack of effect of AAB and AAS extracts on lateral motilities of MDA-MB-231 and MCF-7 cell lines

**Fig. 6:**
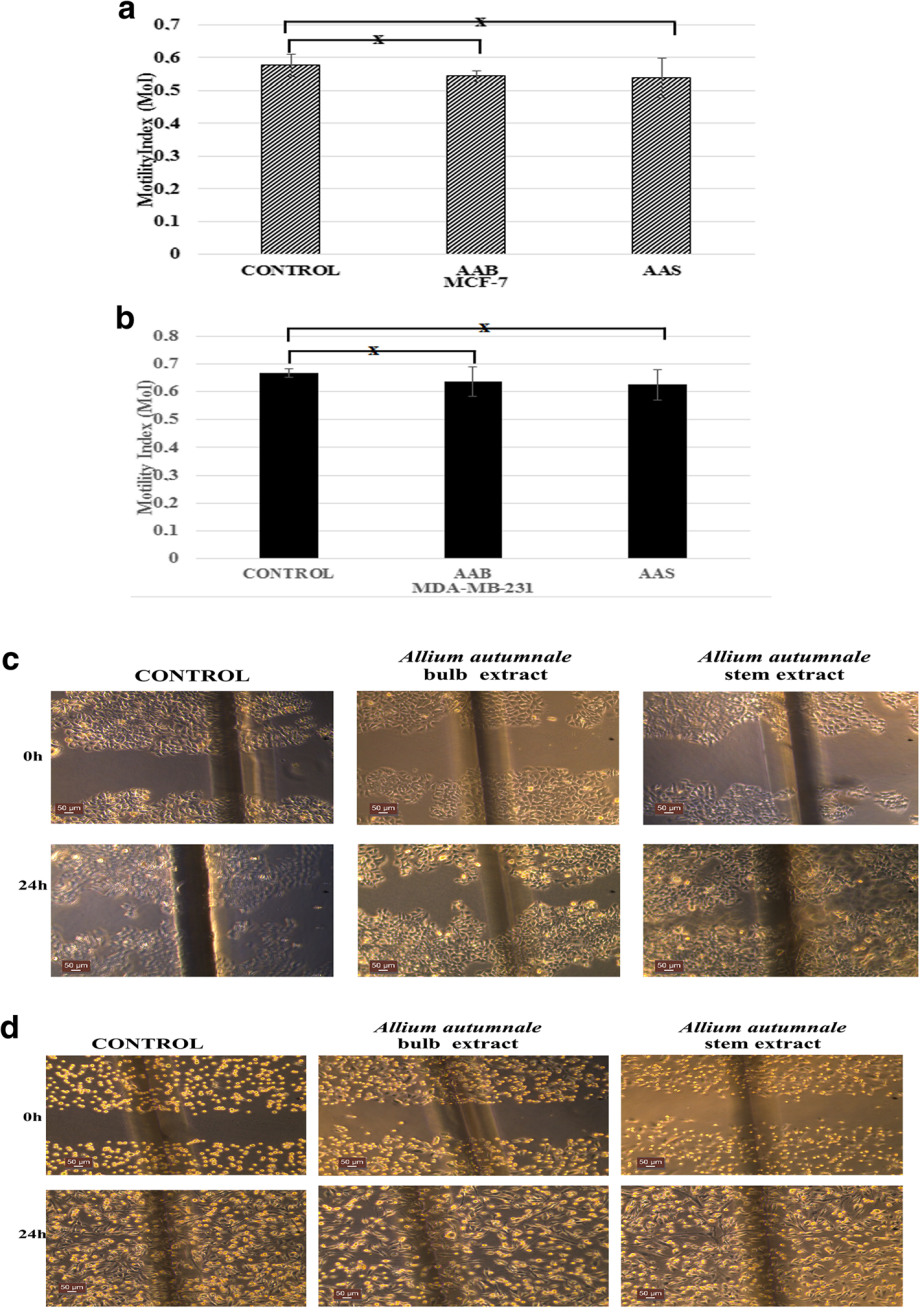
Effects of AAB and AAS on lateral motility of MCF-7 and MDA-MB-231 cells from 24 h incubation. Bar graph showing lateral motility data obtained for MCF-7 (**a**) and MDA-MB-231 (**b**) for 24 h incubation. “x” represents no significant difference compared to the control experiments. Representative inverse light microscope images of AAB and AAS incubation of MCF-7 (**c**) cells and MDA-MB-231 (**d**) which caused no significant effect on lateral motility. Scale bars = 50 μm

The effect of AAB and AAS treatment on lateral motility of both cell lines MCF-7 and MDA-MB-231 were investigated using wound healing assays. Distance of movement in a lateral direction in MCF-7 and MDA-MB-231 cells were determined by using MoI calculation. The concentration of the AAB and AAS extract applied on BCa cells was 312.5 μg/mL as no anti-proliferative or cytotoxic effects were observed on either cell line. AAB and AAS extract treatment (24 h) of MCF-7 cells did not cause any significant effect on lateral motility (MoI control: 0.577 ± 0.034; MoI AAB: 0.0543 ± 0.015, MoI AAS: 0.0537 ± 0.61; *p* > 0.05; *n* = 6) (Fig. 6a, c). Similarly AAB and AAS extract treatment (24 h) of MDA-MB-231 cells had no significant effect on lateral motility (MoI control: 0.667 ± 0.015; MoI AAB: 0.63 ± 0.05; MoI AAS: 0.624 ± 0.03; *p* > 0.05; *n* = 6) (Fig. 6b, d).

### GC-MS analysis of AAB and AAS extracts

**Fig. 7:**
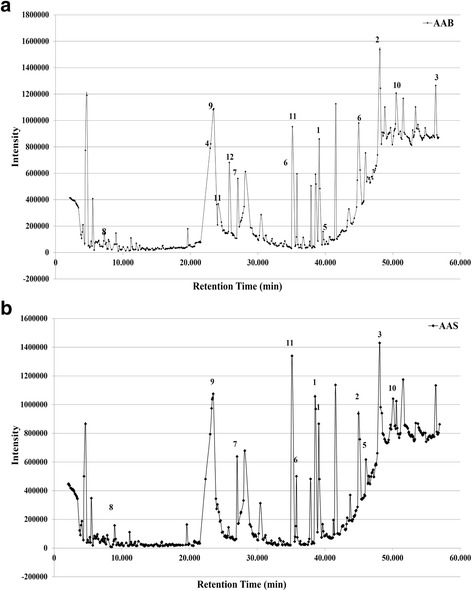
GC-MS chromatogram of compounds from ethanol extract in (**a**) *Allium autumnale* bulb and (**b**) *Allium autumnale* stem. The numbered peaks correspond to the numbers and molecules in Table 1

GC-MS analysis was carried out in order to investigate the presence of any potential anti-cancer or medically important compounds in the composition of AAS and AAB (Fig. 7). GC-MS chromatography analysis of the ethanolic extract of AAB and AAS extract revealed prominent molecules associated with anti-cancer properties (Table 1 and Fig. 7). The most prevailing compounds present in the AAB extract in high quantities, with medical importance are; 9-octadecenoic acid (6.55%), octadecamethylcyclononasiloxane (4.87%), tetrapentacosane (2.43%), l-Isoleucine (6.55%), heptadecanoic acid (0.47%), hexadecanoic acid (1.54%), 1,2-benzenedicarboxylic acid, diethyl ester (1.35%), dimethyltrisulfide (3.31%), (−)-1 L–cyclohex-5-ene-1,3/2,4-tetrol (4.73%), 14-.beta.-h-pregna (1.18%), pentadecanoic acid (1.66%), and quinic acid (1.74%) (Table 1). Furthermore, 9-octadecenoic acid (3,93%), octadecamethylcyclononasiloxane (4.26%), tetrapentacosane (5.69%), heptadecanoic acid (0.68%), hexadecanoic acid (1.16%), 1,2-benzenedicarboxylic acid, diethyl ester (0.19%), dimethyltrisulfide (0.19%), (−)-1 L–cyclohex-5-ene-1,3/2,4-tetrol (10.61%), 14-.beta.-h-pregna (3.56%), and pentadecanoic acid (1.12%) are determined as the major compounds present in the AAS extract with previously revealed, medically important functions (Table 1).

**Table 1:** Compounds detected by GC-MS analysis and biological activities of the identified bioactive compounds from AAB and AAS extract

	Compound Name	*Allium autumnale* bulb % content^a^	*Allium autumnale* stem % content^a^	Biological Activity
1	9-Octadecenoic acid (Z)-, ethyl ester (CAS) Ethyl oleate	6.55%	3.93%	Anti-cancer, anti-proliferative [16–18], anti-oxidantant, anti-microbial [25]
2	Octadecamethyl cyclononasiloxane	4.87%	4.26%	Anti-fungal [26]
3	Tetrapentacosane	2.43%	5.69%	_
4	L-Isoleucine (CAS) Isoleucine, L-	6.55%	_	Plasma glucose lowering amino acid [23]
5	Heptadecanoic acid, ethyl ester (CAS) Ethyl n-heptadecanoate	0.47%	0.68%	Anti-microbial [27]
6	Hexadecanoic acid (CAS) Palmitic acid	1.54%	1.16%	Anti-tumoral [28], anti-microbial, anti-oxidant, decrease blood cholesterol, anti-inflammatory [18]
7	1,2-Benzenedicarboxylic acid, diethyl ester (CAS) Ethyl phthalate	1.35%	2.85%	Anti-cancer [27], anti-microbial, anti-fungal, anti-malarial [17, 18], anti-oxidant, anti-scabies, anti-inflammatory, anti-diabetic [29]
8	Trisulfide, dimethyl (CAS) 2,3,4-Trithiapentane	3.31%	0.19%	Anti-proliferative and apoptotic [24], anti-microbial [30]
9	(−)-1 L–Cyclohex-5-ene-1,3/2,4-tetrol	4.73%	10.61%	Anti-feedant, antibiotic, anti-leukemic [31]
10	14-.Beta.-H-Pregna	1.18%	3.56%	__
11	Pentadecanoic acid	1.66%	1.12%	Anti-microbial, anti-fungal [32]
12	Quinic acid	1.74%	_	Astringent [33]

^a^% peak area relative to GC-MS chromatogram

## Discussion

Anti-cancer effects of several *Allium* species like the traditional garlic, *Allium sativum*, and the closely related *Allium willeanum,* endemic to the island of Cyprus, were previously studied by our team revealing significant anti-cancer properties (O. Isbilen et al., unpublished data). Data obtained from previous studies made these understudied endemic *Allium* species attractive research interests because of their anti-proliferative, cytotoxic and anti-motility properties, leading to our hypothesis that the related species *Allium autumnale* also exhibit similar activity. Several anti-cancer assays carried out on two human breast cancer cell lines, one with strong and one with weak metastatic capacity (MDA-MB-231 and MCF-7, respectively) revealed that the never before studied species, *Allium autumnale,* is associated with anti-proliferative and cytotoxic activities.

Our studies investigated both the bulb and the stem extracts of *Allium autumnale*. The bulb extract (AAB) gave rise to consistently higher cytotoxic and antiproliferative effects on both breast cancer cell lines, with the cytotoxic effects being generally more prominent on the strongly metastatic MDA-MB-231 cell line at higher concentrations than the weakly metastatic MCF-7 cell line over time. This feature is promising when potential therapeutic activity of this species is considered as inhibition of metastatic cancers is clinically more challenging than that of the weakly metastatic ones. The phytochemical profiling of the AAB extract also revealed molecules more consistent with cytotoxic and anti-proliferative activities (Table 1). While the AAB extract demonstrated significant anti-proliferative activity on both tested cell lines with a more robust effect on the MCF-7 cell line, the stem extract (AAS) exhibited lower anti-proliferative activity on both cell types.

The significant anti-proliferative activity of AAB on both tested cell lines, and the lower anti-proliferative activity of AAS, indicates a role that octadecanoic acid content of extracts may play in inhibiting proliferation of BCa cells. Octadecanoic acid, which was previously demonstrated to have anti-proliferative activity [16–18], is found in higher levels in the bulb, compared to the stem extract (Table 1). Overall, MTT experiments demonstrated that both AAB and AAS extracts have more inhibitory effect on the proliferation of MCF-7 breast cancer cells when compared to the MDA-MB-231 cells which tend to proliferate and metastasize faster than the MCF-7 cells.

Trypan blue exclusion assay employed to investigate cytotoxic properties of *Allium* extracts also revealed significant cytotoxic effects on both cell lines albeit at different time points. Treatment with AAB extract demonstrated higher toxicity on MCF-7 cells at early and middle time points (24 and 48 h) and concentrations (1250 μg/mL-2500 μg/mL) whereas a more robust cytotoxic effect on MDA-MB-231 cells was observed at a later time point (72 h). This is likely due to the faster growing, strongly metastatic nature of the MDA-MB-231 cell line.

On the other hand, AAS extract demonstrated more cytotoxic effect on MDA-MB-231 cells compared to the MCF-7 at all tested (24, 48, 72 h) incubation periods specifically at 2500, 5000 and 10,000 μg/mL concentrations. This may likely indicate an alternative mechanism of death experienced by MDA-MB-231 cells as plasma membrane blebs were also observed in this cell line upon exposure to AAS.

Plasma membrane blebs are sign of apoptosis in cells [19]. It was previously shown that 1,2-benzenedicarboxylic acid treatment of the HepG2, MCF-7, NIH3T3 and HaCaT cell lines have shown the presence of plasma membrane blebbing [20]. Higher concentration of 1,2- benzenedicarboxylic acid was found in AAS compared to the AAB (Table 1) where incubation of MDA-MB-231 cell line with AAS caused blebbing likely due to apoptotic mechanisms (Fig. 5). Several studies observed membrane blebbing occurring during locomotion, in instances like embryogenesis [21] and during necrosis especially after free radical exposure and metabolic poisoning [22]. While these are potential causes for bleb formation, the mechanism governing the bleb formation in our model is likely apoptosis mediated as a spike in Caspase-3 activation was observed upon incubation with the extract (data not shown). Interestingly, no blebbing was observed on MCF-7 cells likely because the compound concentration in the extract used was lower than previously published studies [20] or a different mechanism of death is employed in this cell line. Alternatively, the presence or absence of other accompanying molecules in the extract may have caused the blebbing to only be observed in MDA-MB-231 human breast cancer cells and not in MCF-7 cells.

GC-MS analysis carried out on both AAB and AAS extracts have revealed several medically important compounds. Major compounds identified were; 9-octadecenoic acid, hexadecanoic acid, 1,2-benzenedicarboxylic acid and dimethyltrisulfide are with previously shown anti-cancer effects [17, 20, 23, 24]. We hypothesize that at least two of these compounds, 9-octadecenoic acid and 1,2-benzenedicarboxylic acid may directly be involved in the anti-cancer processes we have observed, particularly in preventing proliferation of cancer cells (9-octadecenoic acid) and causing membrane blebs (1,2-benzenedicarboxylic acid).

Lateral motility experiments were carried out by using wound heal assay. Both MCF-7 and MDA-MB-231 breast cancer cells were incubated with AAB and AAS extract. At the end of the 24 h treatment, it was shown that AAB and AAS extract incubation have caused no significant inhibition in cancer cell motility. Interestingly, 9-octadecanamide which was found in high concentration in *Allium willeanum* have an important role in inhibition of lateral motility of MCF-7 and MDA-MB-231 BCa cells (O. Isbilen et al., unpublished data). The lack of 9-octadecanamide in *Allium autumnale* might be the reason for no observed effect on lateral motility of BCa cells.

## Conclusion

Overall, in our present study, screening anti-cancer effects of AAB and AAS extracts revealed significant anti-proliferative and cytotoxic activities associated with the understudied endemic species *Allium autumnale* on model human breast cancer cell lines. Accordingly, active biological molecules obtained from these species could serve as potential anti-cancer agents, either as mono- or combination-therapies, depending on their cellular mechanisms of action on different cancer cell types.

## Abbreviations

AAB: *Allium autumnale* bulb
AAS: *Allium autumnale* stem
BCa: Breast Cancer
CO_2_: Carbon dioxide
DMEM: Dulbecco’s Modified Eagle Medium
FBS: Fetal Bovine Serum
GC-MS: Gas Chromatography Mass Spectroscopy
MTT: Methyl-thiazolyl tertrazolium
WHO: World Health Organization
